# Entamoeba histolytica EHD1 Is Involved in Mitosome-Endosome Contact

**DOI:** 10.1128/mbio.03849-21

**Published:** 2022-04-11

**Authors:** Herbert J. Santos, Yuki Hanadate, Kenichiro Imai, Haruo Watanabe, Tomoyoshi Nozaki

**Affiliations:** a Department of Biomedical Chemistry, Graduate School of Medicine, The University of Tokyo, Tokyo, Japan; b Department of Parasitology, National Institute of Infectious Diseases, Tokyo, Japan; c Cellular and Molecular Biotechnology Research Institute, National Institute of Advanced Industrial Science and Technology, Tokyo, Japan; d Department of Bacteriology, National Institute of Infectious Diseases, Tokyo, Japan; Rutgers—New Jersey Medical School

**Keywords:** EH domain, *Entamoeba histolytica*, endosome, membrane contact site, mitochondrion-related organelles, mitosome

## Abstract

Interorganellar cross talk is often mediated by membrane contact sites (MCSs), which are zones where participating membranes come within 30 nm of one another. MCSs have been found in organelles, including the endoplasmic reticulum, Golgi bodies, endosomes, and mitochondria. Despite its seeming ubiquity, reports of MCS involving mitochondrion-related organelles (MROs) present in a few anaerobic parasitic protozoa remain lacking. Entamoeba histolytica, the etiological agent of amoebiasis, possesses an MRO called the mitosome. We previously discovered several *Entamoeba*-specific transmembrane mitosomal proteins (ETMPs) from *in silico* and cell-biological analyses. One of them, ETMP1 (EHI_175060), was predicted to have one transmembrane domain and two coiled-coil regions and was demonstrated to be mitosome membrane integrated based on carbonate fractionation and immunoelectron microscopy (IEM) data. Immunoprecipitation analysis detected a candidate interacting partner, EH domain-containing protein (EHD1; EHI_105270). We expressed hemagglutinin (HA)-tagged EHD1 in E. histolytica, and subsequent immunofluorescence and IEM data indicated an unprecedented MCS between the mitosome and the endosome. Live imaging of a green fluorescent protein (GFP)-EHD1-expressing strain demonstrated that EHD1 is involved in early endosome formation and is observed in MCS between endosomes of various sizes. *In vitro* assays using recombinant His-EHD1 demonstrated ATPase activity. MCSs are involved in lipid transfer, ion homeostasis, and organelle dynamics. The serendipitous discovery of the ETMP1-interacting partner EHD1 led to the observation of the mitosome-endosome contact site in E. histolytica. It opened a new view of how the relic mitochondria of *Entamoeba* may likewise be involved in organelle cross talk, a conserved feature of mitochondria and other organelles in general.

## INTRODUCTION

Membrane contact sites (MCSs) mediate communication and exchanges between membrane-bound compartments by the assembly of protein-protein or protein-lipid tethers, which maintains distancing of 30 nm between interacting membranes. MCSs have been found in almost every pair of organelles (1), most of which involve the endoplasmic reticulum (ER), as its membrane spans a network that interacts with the plasma membrane, and other organellar membranes, such as those of the Golgi apparatus, lysosomes, endosomes, lipid droplets, peroxisomes, and mitochondria (2). MCSs are also reported between other organelle pairs, including the peroxisomes and lipid droplets and the mitochondria and vacuoles/endosomes/lysosomes, plasma membrane, lipid droplets, and peroxisomes, notwithstanding the contact sites between the inner and outer mitochondrial membranes (3). These contact sites mostly harbor proteins involved in lipid metabolism and transport, making them hubs of lipid transfer between interacting membranes. However, other processes associated with MCSs have been reported and they include ion transport and homeostasis, apoptosis (1), and endosomal (4) and mitochondrial (5) fission.

We recently identified several key molecules that facilitate membrane contact sites in the mitosomes, endosomes, and Golgi apparatus of Entamoeba histolytica and aimed to study their roles and possible link to the parasitic nature of this amoeba. E. histolytica is an anaerobic unicellular protozoan parasite that infects the large intestine of humans and causes amebiasis, a disease characterized by diarrhea, which is a major cause of death in children worldwide. Millions of individuals are infected, mostly in developing countries, and the disease causes an estimated 100,000 deaths annually (6). Infection begins by the ingestion of infectious cysts, which are resistant to the acidic environment of the stomach; the cysts then pass through the small intestine and undergo excystation within the terminal ileum or colon, to the trophozoite stage. Trophozoites reproduce and encyst within the colon, where they are released in the environment via excretion of feces, thus completing one cycle of fecal-oral transmission (6). Invasive amoebic trophozoites destroy the mucoepithelial barrier of the host intestinal tract, inducing mucus overproduction, inflammation, and dysentery. This can lead to the formation of extraintestinal abscesses, particularly in the liver (amoebic liver abscess), lungs, and brain. The virulence of this parasite is due to its ability to inflict damage to host cells and tissues by parasite attachment to colonic epithelial cells, by protease secretion to damage host cells and evade host immune response, and by ingestion of host cells via phagocytosis and trogocytosis. These processes involve intracellular trafficking and interorganellar cross talk, underscoring the role of vesicular transport and MCSs not only in parasite biology but also in its virulence and pathogenesis.

Like other anaerobic parasitic protozoans, E. histolytica lacks canonical mitochondria and instead has a highly divergent mitochondrion-related organelle (MRO) called the mitosome. *Entamoeba* mitosomes contribute to parasitism (7) due to a compartmentalized sulfate activation pathway that leads to the formation of cholesteryl sulfate in the cytosol. This molecule induces stage conversion from the trophozoite to cyst form (8), a process that is essential for maintaining the parasite’s life cycle and mode of disease transmission. Apart from mitosomes, other amoebic organelles, such as the ER and the Golgi apparatus, also show less defined structural and compositional features compared with model organisms; however, they have been shown to contain orthologs of established endomembrane proteins (9–11). Our knowledge of MCSs in *Entamoeba* is extremely limited, with only the mitosomal membrane proteins ETMP30 (reported to interact with a Golgi-localized protein secretory pathway calcium ATPase) and EHI_099350 (reported to have dual localization in the mitosomes and the ER) (12–14) having been identified as mediators of interorganellar contact so far. What other molecules participate in tethering of these compartments and what roles these contact sites play in the cell are still unknown, making it imperative to dissect amoebic MCSs. These past observations point to the fact that mitosomes, although highly degenerate, are able to interact with other organelles in the cytoplasm, and such contacts often utilize lineage-specific membrane proteins. Here, we identified another mitosomal membrane protein, ETMP1, which interacts with a protein containing a C-terminal Eps15 homology domain (EHD), a member of the EHD protein superfamily involved in various endocytic processes. Studies on E. histolytica organelle interaction via the endocytic transport mechanism have accumulated over several decades, including those reporting proteins involved in cargo sorting regulation and endosome dynamics, such as Rab GTPases (15), and ESCRT (endosomal sorting complex required for transport) proteins (16, 17). However, there are so far no reports on whether these molecules take part in MCSs between endosomes and other parts of the cell.

## RESULTS

### EHI_175060 is a lineage-specific mitosomal membrane protein.

**FIG 1:**
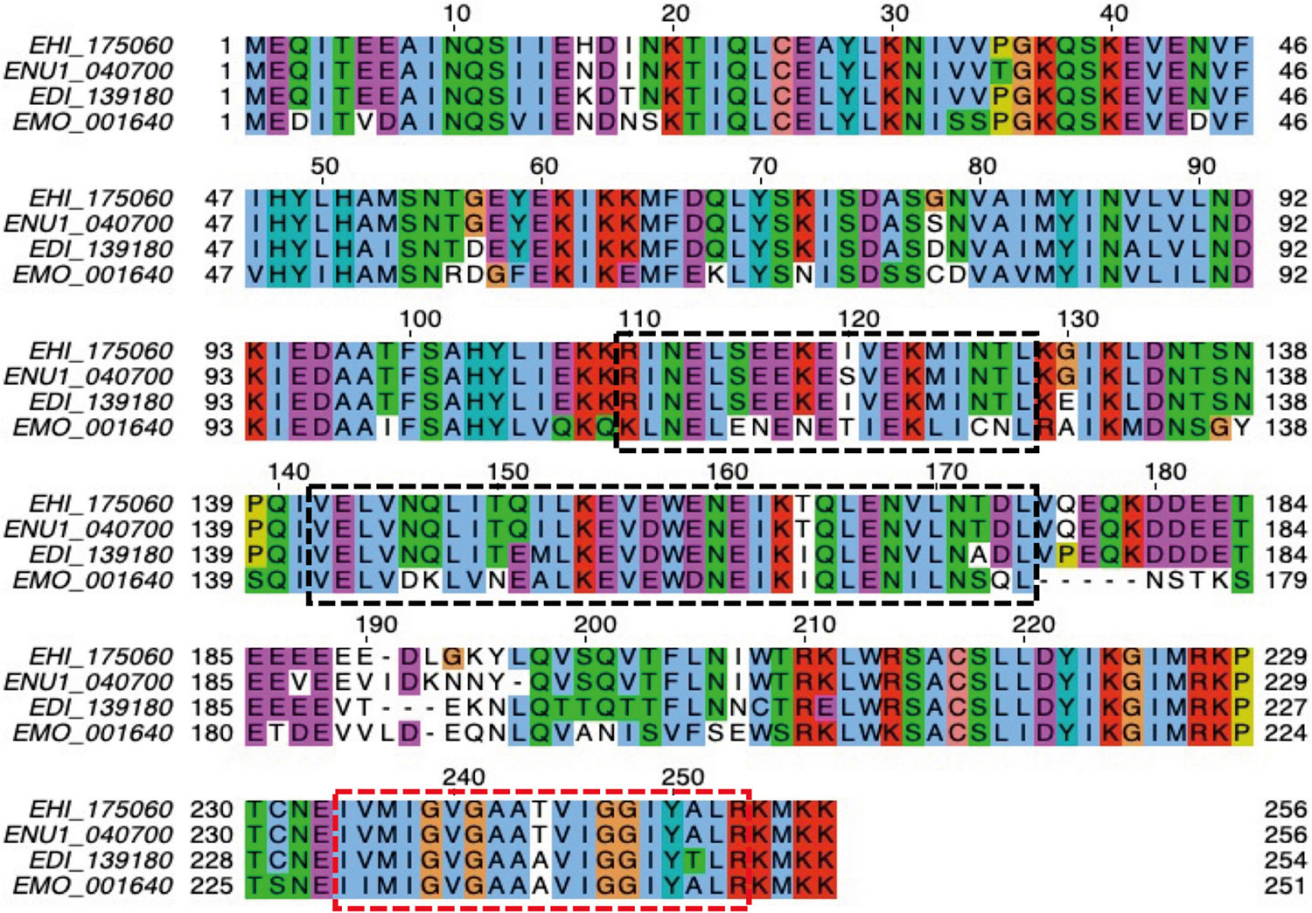
Multiple sequence alignment of ETMP1 orthologs in *Entamoeba*. Amino acid sequences of orthologs in E. histolytica (EHI_175060), *E. nuttalli* (ENU1_040700), *E. dispar* (EDI_139180), and *E. moshkovskii* (EMO_001640) were aligned using MAFFT (67) and displayed using Jalview (68). The hydrophobic, positively charged, negatively charged, polar, cysteine, glycine, proline, and aromatic residues are indicated in blue, red, magenta, green, pink, orange, yellow, and cyan, respectively. Dashed black boxes show the coiled-coil domains predicted by DeepCoil (64), while the dashed red box indicates the transmembrane region predicted by our TMD prediction tool (12).

Our group previously searched for transmembrane domain-containing mitosomal proteins using a previously developed prediction pipeline (12) which sought for proteins that could be lineage-specific receptors, channels, enzymes, and components of the import machinery or otherwise uncharacterized complexes on the outer and inner membranes of Entamoeba histolytica mitosomes. This resulted in the prediction of 25 protein candidates. Like the other 24 proteins in the list (12), EHI_175060 is unique to the lineage *Entamoeba*. Figure 1 shows a multiple-sequence alignment of the protein sequence of EHI_175060 with that of its orthologs in other *Entamoeba* species (Entamoeba moshkovskii, E. dispar, and E. nuttalli). The protein has a predicted molecular mass of 29.5 kDa, and it contains two coiled-coil domains in the middle portion and a single transmembrane domain near the carboxyl terminus. It also lacks a predictable canonical N-terminal targeting sequence. Based on these characteristics, we name EHI_175060 *Entamoeba*-specific transmembrane mitosomal protein 1 (ETMP1).

### ETMP1 is localized to mitosomal membranes.

**FIG 2:**
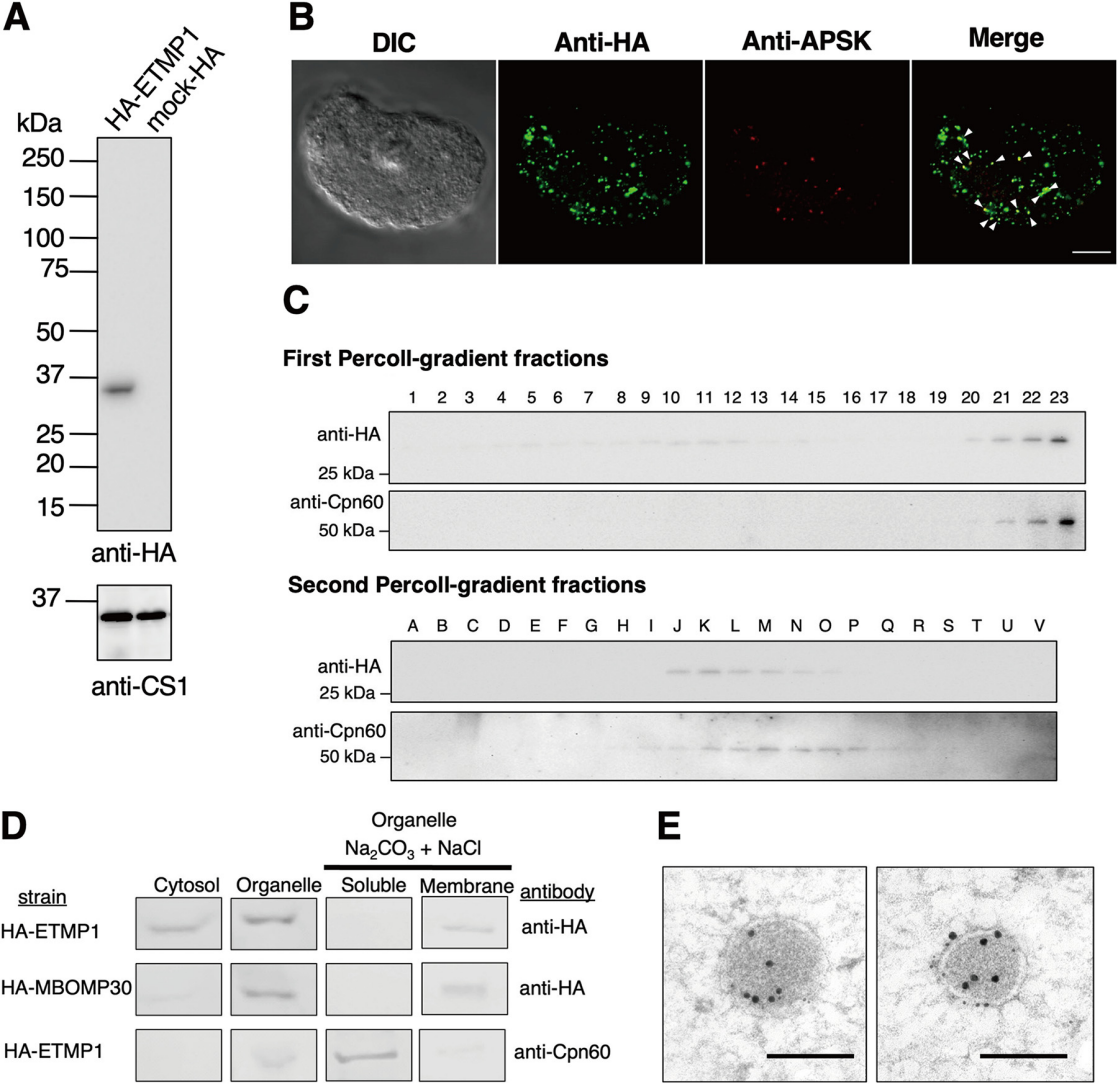
Expression and localization of HA-ETMP1 in E. histolytica trophozoites. (A) Approximately 30 μg protein from whole-cell lysates of HA-ETMP1 and mock control (pEhEx-HA) strains were separated by SDS-PAGE and subjected to anti-HA immunoblot analysis (top). The 33-kDa band corresponds to the predicted molecular mass of HA-ETMP1. As a loading control, cysteine synthase 1 (CS1) was probed using anti-CS1 antibody (bottom). (B) Immunofluorescence analysis of HA-ETMP1-expressing trophozoites, double stained with anti-HA (green) and anti-APSK (red). White arrowheads in the merged panel point to colocalization of anti-HA and anti-APSK signals. Bar = 10 μm. (C) Fractionation of HA-ETMP1 by discontinuous Percoll gradient ultracentrifugation. Homogenate of HA-ETMP1 was separated by density against a Percoll gradient. Approximately 15 μL of fractions collected from the first (1 to 22) and second (A to V) ultracentrifugation steps was separated by SDS-PAGE followed by immunoblot analysis with anti-HA and anti-Cpn60 antibodies, respectively. (D) Anti-HA and anti-Cpn60 immunoblot profiles of subcellular fractionation, including alkaline carbonate-treated organelle-rich fractions of HA-ETMP1 and HA-MBOMP30 (mitosome membrane control). (E) Representative immunoelectron micrographs of 15-nm anti-APSK–gold-labeled mitosomes of HA-ETMP1, costained with 5-nm anti-HA–gold. Bar = 200 nm.

To validate the predicted localization of ETMP1, we expressed an amino-terminally hemagglutinin (HA)-tagged fusion protein, HA-ETMP1, in amoebic trophozoites and confirmed protein expression by Western blotting analysis. The anti-HA immunoblot showed a single band corresponding to the expected molecular mass of HA-ETMP1 (Fig. 2A). We then analyzed the localization of the protein by immunofluorescence assay (IFA) (Fig. 2B). Costaining of an HA-ETMP1-expressing strain using anti-HA antibody and anti-adenosine-5′-phosphosulfate kinase (APSK; EHI_179080; a mitosomal matrix enzyme involved in sulfate activation) antiserum revealed good colocalization of the HA-tagged protein to mitosomes containing APSK. This is supported by the Pearson correlation *R* value, which ranges from 0.31 to 0.59, suggesting that HA-ETMP1 is localized to mitosomes (Fig. S1A). Furthermore, we also performed Percoll-gradient fractionation of HA-ETMP1 homogenate and found that fractions containing HA-ETMP1 showed broad distribution in the first ultracentrifugation, suggesting some proteins are localized to the cytosol/lighter fractions. However, HA-ETMP1 also exists in the bottom fractions which overlap those that contain chaperonin 60 (Cpn60; EHI_178570; a chaperone protein and canonical mitochondrial matrix marker). The cofractionation of HA-ETMP1 to mitosomes was suggested by the anti-HA and anti-Cpn60 immunoblots of both the first and second ultracentrifugation (Fig. 2C). We also performed subcellular fractionation followed by carbonate treatment, to further assess the localization, as well as membrane integration of HA-ETMP1. The fractionation profile of HA-ETMP1 after immunoblot analysis (Fig. 2D, top) showed that it is present in both cytosolic and organelle fractions. Also, it was clearly demonstrated that the HA-ETMP1 contained in the organellar membrane-enriched fraction is integrated into organellar membranes, as it was retained in the particulate fraction after carbonate treatment, similar to MBOMP30-HA (Fig. 2D, middle), a positive control for mitosomal membrane protein. These carbonate fractionation profiles contrast with that of the soluble mitosomal matrix protein marker Cpn60, as shown by the blot stained with anti-Cpn60 antiserum (Fig. 2D, bottom).

10.1128/mbio.03849-21.8 FIG S1 (A) Analysis of representative immunofluorescence assay (IFA) images. (Left to right) Merged image of HA-ETMP1 trophozoite, double stained with anti-HA antibody (green) and anti-APSK antiserum (red); plot of green and red fluorescence intensity across the line drawn on the merged micrograph using ImageJ (59); scatterplot of immunofluorescence image generated using the “Colocalization” tool of the Zen software (Carl Zeiss, Germany). The Pearson correlation coefficient (*R*) was computed using the same software. (B) (Left) HA-EHD1 merged IFA images from the main text stained with anti-HA and anti-APSK (top), anti-Vps26 (second from top), anti-PNT (third from top), and anti-Rab11b (bottom). (Middle) Plots of green (anti-HA) and red (anti-APSK/anti-Vps26/anti-PNT/anti-Rab11b) across the line drawn on the merged image obtained using ImageJ (59). (Right) Scatterplots generated using the “Colocalization” tool of the Zen software (Carl Zeiss, Germany). The indicated *R* values were computed using the same software. (C). Representative IFA double-staining images of mock-HA trophozoites. Anti-HA signals are green, while anti-APSK, anti-Vps26, anti-PNT, and anti-Rab11b signals are red. Bar = 10 μm. Download FIG S1, TIF file, 1.8 MB. Copyright © 2022 Santos et al. 2022 Santos et al. https://creativecommons.org/licenses/by/4.0/ This content is distributed under the terms of the Creative Commons Attribution 4.0 International license.

We also performed immunoelectron microscopy analysis, and the results indicated that HA-ETMP1 is localized to the mitosome membranes, as anti-HA–gold particles were found along the periphery of the APSK-labeled mitosomes (Fig. 2E). Particle distribution analysis of the gold-conjugated antibodies revealed a significant difference in the staining of mitosomes (368 ± 279/μm^2^) compared to cytosol (22.3 ± 9.25/μm^2^) by anti-HA–gold. The distribution of the mitosomal marker APSK as detected by the gold–anti-APSK particles was also significantly higher in mitosomes (192 ± 98.1/μm^2^) than in the cytosol (0.984 ± 0.817/μm^2^). Statistical significance in both data sets was analyzed using two-tailed Welch's unequal variance *t* test (*n* = 17, *P < *0.0001). Overall, these data provide evidence of mitosomal membrane localization of ETMP1.

### ETMP1 is essential, and its overexpression causes a drastic growth defect.

**FIG 3:**
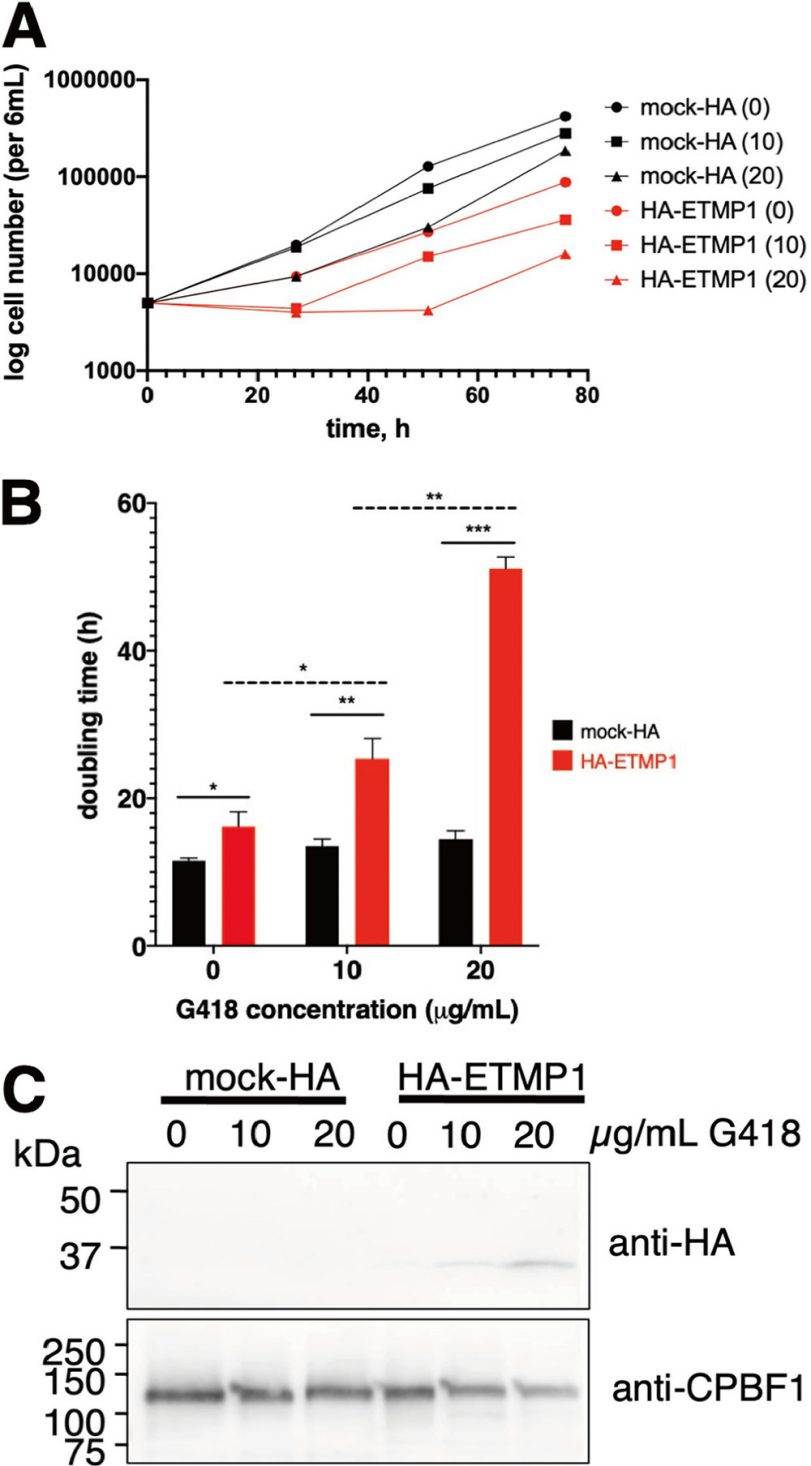
Effect of overexpression on the growth of HA-ETMP1 strain. (A) Representative growth curves showing cell numbers of HA-ETMP1 (red) and mock-HA (black) strains cultivated in BI-S-33 medium containing 0, 10, and 20 μg/mL G418, plotted against time. Growth curves from the other two experiments are shown in Fig. S2A. (B) Doubling time of HA-ETMP1 (red) and mock-HA (black) calculated at various concentration of G418. Statistical significance was analyzed using Student's *t* test. *n* =3. *, *P* < 0.05; **, *P* < 0.005; ***, *P* < 0.0005. (C) Western blot analysis of whole-cell lysates of HA-ETMP1 and mock-HA grown in medium containing 0, 10, and 20 μg/mL G418. Top and bottom panels show anti-HA and anti-CPBF1 (loading control) immunoblots, respectively.

We made several attempts at silencing the *etmp1* gene by small-RNA transcriptional interference, all of which failed, as the transformants did not survive drug selection, suggesting its essentiality to the parasite. We also observed a lower growth rate in HA-ETMP1 expressors than in the empty vector transfected control (referred to here as mock-HA) (*n* = 3). Analysis of growth kinetics of the two strains at various concentrations of Geneticin (G418) suggested a dose-dependent effect of drug concentration on the growth of amoebic trophozoites (Fig. 3A; Fig. S2A), doubling time (Fig. 3B), and protein expression (Fig. 3C). The doubling time of HA-ETMP1 strain was significantly higher (16.2 ± 2.0 h, 25.4 ± 2.8 h, and 51.1 ± 1.6 h) than the mock-HA strain (11.5 ± 0.4 h, 13.5 ± 0.9 h, and 14.5 ± 1.1 h) when maintained using culture medium supplemented with 0, 10, and 20 μg/mL G418, respectively (Fig. 3B). Likewise, doubling time was significantly higher in the HA-ETMP1 strain as the G418 concentration was increased from 0 to 10 μg/mL (*P = *0.0015) and 10 to 20 μg/mL (*P = *0.00065) (Fig. 3B). Statistical significance was analyzed using Student's *t* test.

10.1128/mbio.03849-21.9 FIG S2 (A) Growth curves of HA-ETMP1 (red) and mock-HA (black) maintained in medium supplemented with 0, 10, and 20 μg/mL G418. Results of two additional independent experiments performed 1 week apart are shown. (B) Anti-HA immunoprecipitation of HA-EHD1 and mock-HA control. Western blot analysis of various IP fractions probed using anti-HA antibody (left). Silver-stained SDS-PAGE gel showing separated protein bands from eluted IP samples. Black boxes indicate regions excised and submitted for subsequent protein sequencing analysis (right). Download FIG S2, TIF file, 0.8 MB. Copyright © 2022 Santos et al. 2022 Santos et al. https://creativecommons.org/licenses/by/4.0/ This content is distributed under the terms of the Creative Commons Attribution 4.0 International license.

### ETMP1 interacts with EH-domain containing proteins.

**FIG 4:**
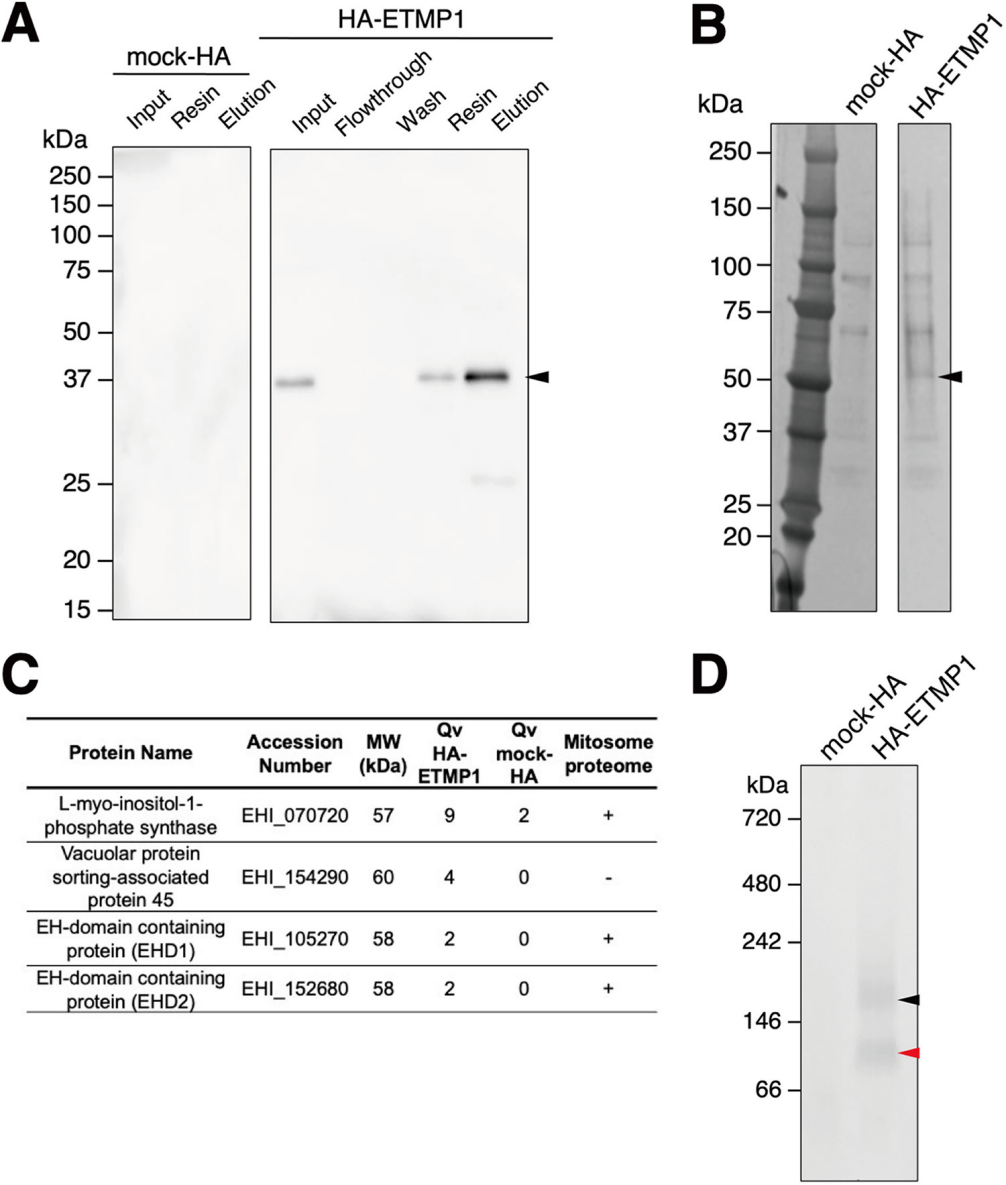
Anti-HA bead immunoprecipitation (IP) of mock-HA and HA-ETMP1 strains. (A) Western blot analysis using anti-HA antibody of the cell lysates and various IP fractions of mock-HA (left) and HA-ETMP1 (right). A black arrowhead indicates the position of HA-tagged ETMP1 (33 kDa). (B) Silver-stained-SDS-PAGE gel of IP eluates of mock-HA and HA-ETMP1 strains. A black arrowhead points to a specific ∼55-kDa band unique to HA-ETMP1. (C) Enriched or exclusively detected proteins in the ∼55-kDa excised gel band from HA-ETMP1 IP eluate compared to that of mock-HA control IP eluate by liquid chromatography-tandem mass spectrometry (LC-MS/MS) sequencing analysis. MW, predicted molecular weight; Qv, quantitative value (normalized total spectra). The presence of the detected proteins in the previously published mitosome proteome data (18) was analyzed, and the results are listed in the last column (+, present; −, absent). (D) Total cell lysates of mock-HA and HA-ETMP1 were separated by BN-PAGE, followed by anti-HA Western blot analysis. Black and red arrowheads indicate the ∼180-kDa and ∼90-kDa complexes, respectively, that contain HA-ETMP1.

To shed light on the function of ETMP1, we next attempted to identify its interacting partner(s) by immunoprecipitation (IP). Anti-HA agarose beads were used to immunoprecipitate the bait protein together with its binding partner(s) from the organelle-rich fractions of HA-ETMP1-expressing and mock-HA control strains. Western blotting with anti-HA antibody confirmed successful binding to and elution from HA-ETMP1 with respect to the anti-HA beads (Fig. 4A). Silver staining of the SDS-PAGE gel containing HA peptide-eluted fractions revealed a band corresponding to approximately 55 kDa that is uniquely precipitated in the HA-ETMP1 strain (absent in the mock-HA control) (Fig. 4B). Protein sequencing analysis by mass spectrometry followed by differential comparison of quantitative values (QVs), normalized with unweighted spectrum counts between HA-ETMP1 and mock-HA control, identified interacting partners of ETMP1 (Fig. 4C). Using a QV cutoff of >2.0 in the HA-ETMP1 strain over the mock-HA sample yielded four candidates, three of which were exclusively detected in the eluted IP fraction of HA-ETMP1. Also, three of the four candidates were identified in the mitosome proteome that was previously published (18), namely, l-myo-inositol-1-phosphate synthase (EHI_070720), EH domain (EHD)-containing protein 1 (annotated as receptor-mediated endocytosis protein; EHI_105270), and its close homolog, EHD2 (EHI_152680).

We also performed blue native PAGE (BN-PAGE) analysis to assess whether ETMP1 is part of a protein complex. Anti-HA immunoblot analysis of BN-PAGE-run samples indicated that HA-ETMP1 forms complexes of about 90 kDa and 180 kDa (Fig. 4D). Protein sequencing analysis of the excised silver-stained BN-PAGE bands containing these two complexes identified numerous proteins. Similarly, we set a cutoff value of >2.0, and the list of proteins is in Table S1A to C. Notably, EHD1 and its close homolog EHD3 (97% identical) were identified in both the 90- and 180-kDa complex bands. Thus, we regarded EHD1 as one of the potential interacting partners of ETMP1.

10.1128/mbio.03849-21.10 TABLE S1 (A) Exclusively detected proteins in the ∼180-kDa excised BN-PAGE gel band from HA-ETMP1 compared to that of the mock-HA control by LC-MS/MS sequencing analysis. (B) Detected proteins in the ∼180-kDa excised BN-PAGE gel band enriched in HA-ETMP1 compared to that of mock-HA control by LC-MS/MS sequencing analysis (Qv HA/ETMp1/mock-HA ≥ 2.0.) (C) Proteins detected exclusively in the ∼90-kDa excised BN-PAGE gel band enriched in HA-ETMP1 compared to that of the mock-HA control by LC-MS/MS sequencing analysis. (D) Proteins identified in the 55- to 58-kDa excised gel band of HA-EHD1 and mock-HA IP eluate samples by LC-MS/MS analysis. (E) Proteins identified in the 30- to 33-kDa excised gel band of HA-EHD1 and mock-HA IP by LC-MS/MS analysis. MW, predicted molecular weight; QV, quantitative values (normalized total spectra). Download Table S1, XLSX file, 0.02 MB. Copyright © 2022 Santos et al. 2022 Santos et al. https://creativecommons.org/licenses/by/4.0/ This content is distributed under the terms of the Creative Commons Attribution 4.0 International license.

### EHD1 is an ETMP1-interacting protein that is localized to mitosomes and to vesicles of various sizes.

**FIG 5:**
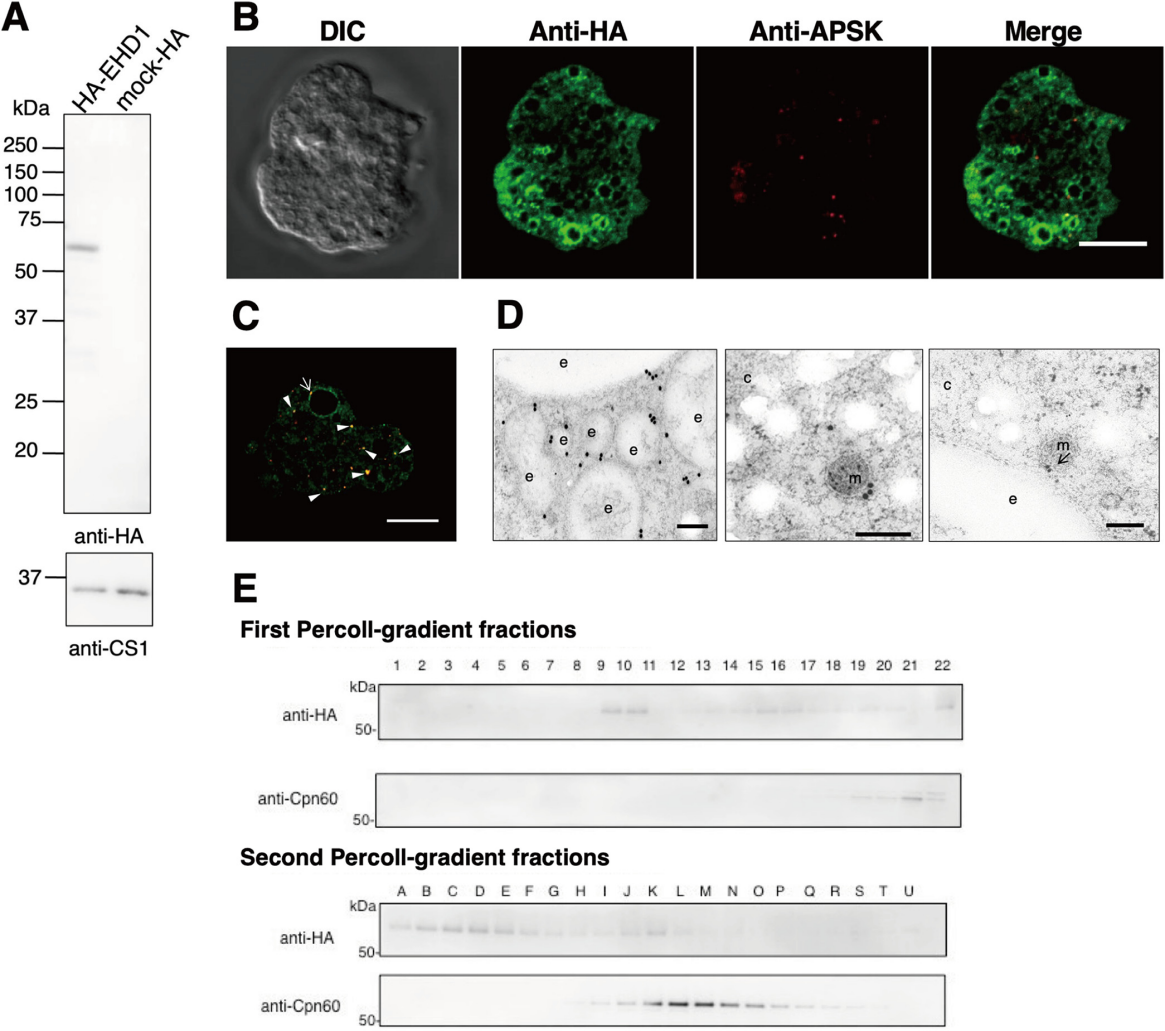
HA-EHD1 expression in E. histolytica trophozoites. (A) Anti-HA immunoblot analysis of approximately 30 μg total cell lysates of mock-HA and HA-EHD1 shows a 61-kDa band corresponding to HA-tagged EHD1 (top). CS1, detected by anti-CS1 antiserum, was used as a loading control (bottom). (B and C) Representative immunofluorescence images of fixed HA-EHD1-expressing cells double-stained with anti-HA (green) and anti-APSK (red) antibodies. The arrow and arrowheads indicate proximity and colocalization between anti-HA and anti-APSK signals, respectively. Bar = 10 μm. (D) Representative immunoelectron micrographs of HA-EHD1 trophozoites, double stained with 5-nm anti-HA–gold and 15-nm anti-APSK–gold. Bar = 200 nm. c, cytosol; e, endosome; m, mitosome. An arrow points to the structure where the membranes of the mitosome and endosome are in close contact. The mitosome in the right panel was identified by its discrete double-membrane structure and highly electron-dense matrix. (E) Percoll gradient fractionation of HA-EHD1 followed by Western blotting analysis using anti-HA and ant-Cpn60 antibodies.

We expressed EHD1 in amoeba trophozoites with an HA tag at the amino terminus, as confirmed by the anti-HA immunoblot result showing a band corresponding to the expected molecular mass of HA-EHD1 (∼61 kDa) (Fig. 5A). To analyze and confirm the mitosomal localization of EHD1, we performed double-staining IFA on the HA-EHD1-expressing strain with anti-HA antibody and anti-APSK antiserum. We observed that the anti-HA signal is mostly localized to the membrane of vesicles of various sizes (Fig. 5B). We also noticed a few punctate anti-HA signals which colocalized with the anti-APSK mitosome marker (Fig. 5C, arrowheads). Although minimal colocalization between anti-HA and anti-APSK signals was observed (Pearson correlation *R* value range of −0.12 to 0.18), some anti-APSK signals were notably seen near the vesicle membranes marked with HA-EHD1 (Fig. 5C, arrow). A representative analysis of anti-HA and anti-APSK signal colocalization is shown in Fig. S1B. Immunoelectron analysis (Fig. 5D) corroborated the IFA observations, as we observed mostly vesicular membrane staining of anti-HA–gold particles (Fig. 5D, left), with occasional signals on mitosomal membranes (middle), some of which showed close proximity to vesicular membranes (right). Furthermore, immunoblot analysis of Percoll gradient fractions indicated wide distribution of HA-EHD1 across various densities, mostly in fractions 9 to 10 and with weaker intensity in fractions 12 to 22 of the first ultracentrifugation and in fractions A to N in the second ultracentrifugation (Fig. 5E), validating the microscopic observations of HA-EHD1 vesicular and mitosomal localization.

**FIG 6:**
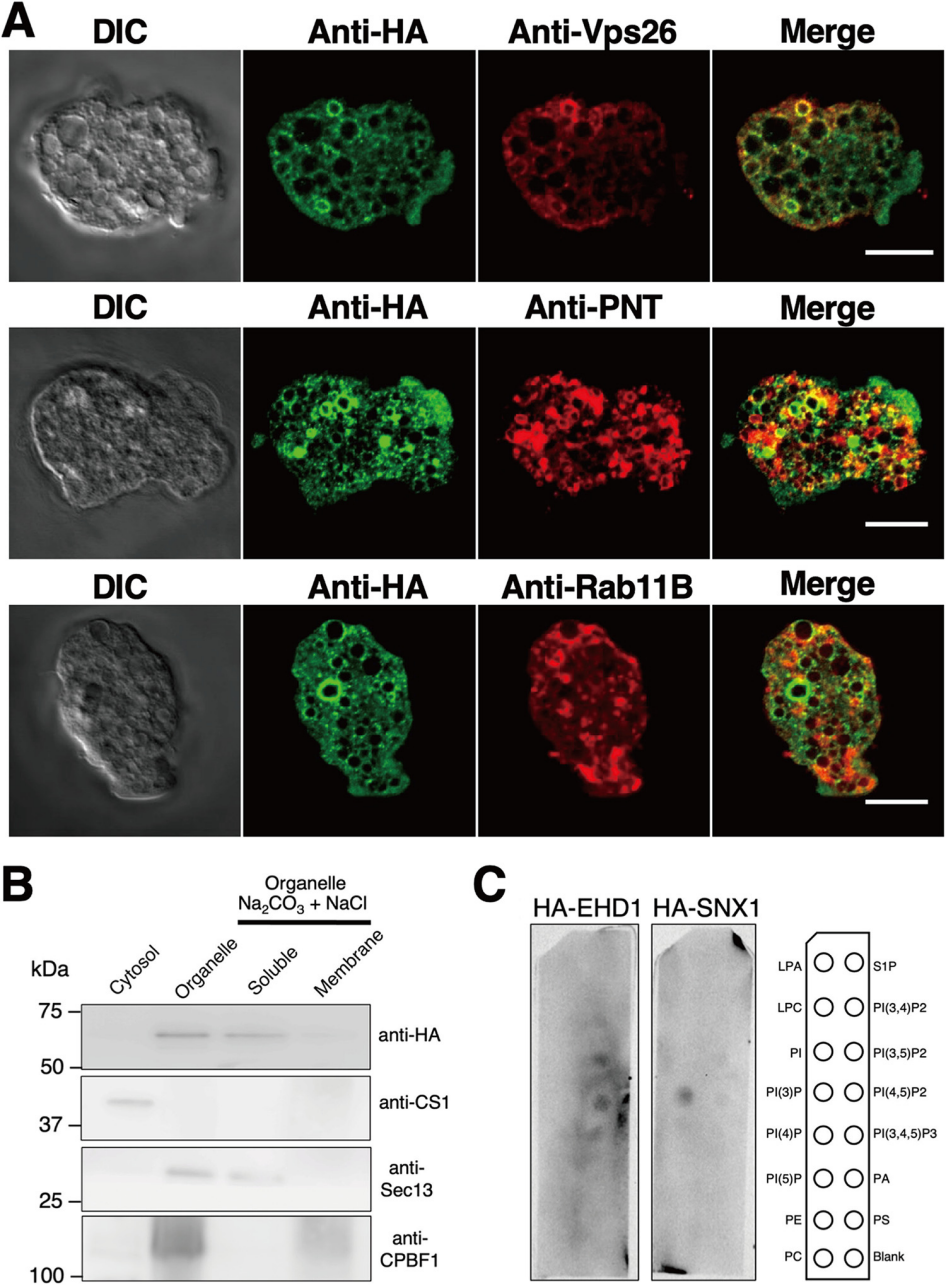
Association of HA-EHD1 with the E. histolytica membranes. (A) Colocalization analysis of HA-EHD1 with various endosomal markers. Representative IFA images of HA-EHD1 costained with anti-HA (green) and anti-vacuolar protein sorting 26 (Vps26 [red, top]), anti-pyridine nucleotide transhydrogenase (PNT [red, middle]), and anti-Rab11B (red, bottom). (B) Immunoblot analysis of carbonate fractionation assay of the HA-EHD1 organelle-rich fraction using (from top to bottom) anti-HA, anti-CS1 (cytosolic protein control), anti-Sec13 (peripheral membrane protein control), and anti-CPBF1 (integral membrane protein control). (C) Lipid overlay assay of HA-EHD1 and HA-SNX1 (PI3P binding protein control). The membrane strips contain 100 pmol of the following lipids per spot: lysophosphatidic acid (LPA), lysophosphocholine (LPC), phosphatidylinositol (PtdIns), phosphatidylinositol (3)-phosphate [PI(3)P], phosphatidylinositol (4)-phosphate [PI(4)P], phosphatidylinositol (5)-phosphate [PI(5)P], phosphatidylethanolamine (PE), phosphatidylcholine (PC), sphingosine 1-phosphate (S1P), phosphatidylinositol (3,4)-bisphosphate [PI(3,4)P2], phosphatidylinositol (3,5)-bisphosphate [PI(3,5)P2], phosphatidylinositol (4,5)-bisphosphate [PI(4,5)P2], phosphatidylinositol (3,4,5)-trisphosphate [PI(3,4,5)P3], phosphatidic acid (PA), and phosphatidylserine (PS).

As the majority of the signals of HA-EHD1 appear on vesicles, we next characterized the vesicles containing HA-EHD1 by performing costaining IFA using anti-HA antibody and one of the following antisera: anti-vacuolar-protein sorting 26 (Vps26), anti-pyridine nucleotide transhydrogenase (PNT), and anti-Rab11B. Most of the anti-HA-stained vesicles were colocalized with anti-Vps26- rather than anti-PNT- and anti-Rab11B-stained vesicles (Fig. 6A), as supported by the Pearson correlation *R* value ranges of 0.22 to 0.37 for anti-Vps26, −0.16 to 0.19 for anti-PNT, and −0.12 to 0.01 for anti-Rab11B. Colocalization analyses of representative IFA images are shown in Fig. S1B, while representative IFA double-staining images of mock-HA trophozoites are shown in Fig. S1C. Vps26 is a retromer complex component and is a marker of endosomes/phagosomes in E. histolytica (19, 20). PNT is localized to the membrane of numerous vesicles/vacuoles, including lysosomes and phagosomes (21), while Rab11B was demonstrated to partially colocalize with late endosomes (22). Together, these data suggest that EHD1 is mostly localized in endosomal membranes which may contain Vps26 and to some extent PNT, but not Rab11B.

### HA-EHD1 is weakly associated with organellar membranes and preferentially binds to PI(3,5)P_2_ and PI(4,5)P_2_.

We performed subcellular fractionation of the HA-EHD1-expressing strain homogenate. Based on the anti-HA immunoblots, HA-EHD1 was exclusively contained in the organelle fraction, in contrast to the anti-CS1 profile, which represents the cytosolic fraction (Fig. 6B). Next, we also assessed membrane integration of HA-EHD1 by carbonate treatment of the organelle-enriched fraction. Results of the immunoblots showed that HA-EHD1 is not membrane bound, in contrast to the lysosomal membrane protein marker CPBF1 (Fig. 6B). Instead, the profile is similar to that of the blot immunostained with an antiserum targeting Sec13, a peripheral ER membrane protein (Fig. 6B). This suggests that HA-EHD1 is not organellar-membrane integrated but rather is weakly organellar-membrane associated.

To validate and characterize the phospholipid binding capacity of EHD1, we carried out a lipid overlay assay using lysates of HA-EHD1 and HA-SNX1 (phosphoinositol-3-phosphate binding protein control). Results indicated preferential binding of HA-EHD1 to phosphoinositide diphosphates, specifically phosphatidylinositol 3,5-bisphosphate PI(3,5)P_2_ and PI(4,5)P_2_ (Fig. 6C).

### Overexpression of HA-EHD1 demonstrated enhanced MVB formation.

**FIG 7:**
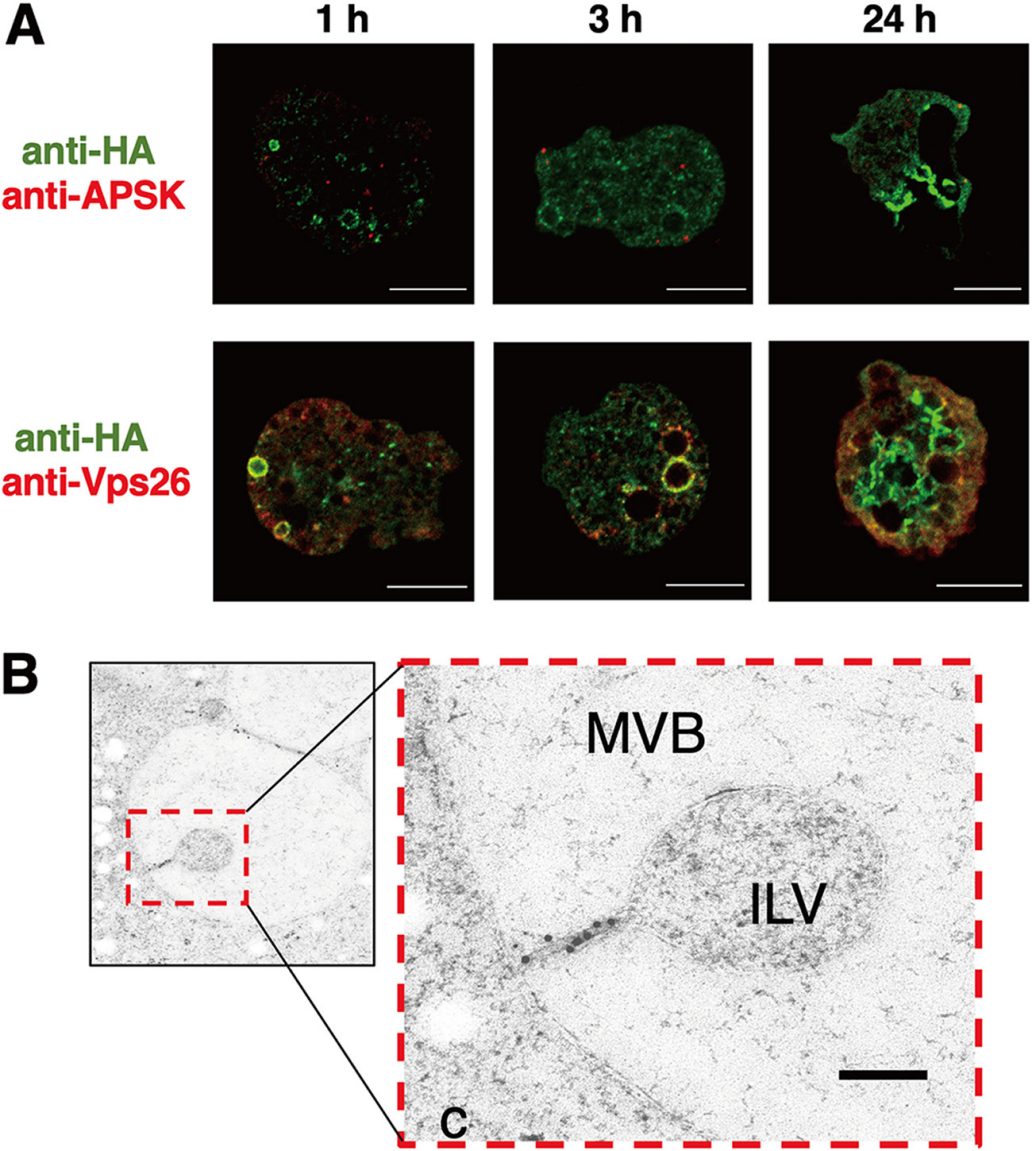
Involvement of HA-EHD1 in multivesicular body formation. (A) Representative anti-HA antibody and anti-APSK antiserum (top) or anti-Vps26 antiserum (bottom) double-staining IFA images of trophozoites that expressed HA-EHD1 trophozoites after 1, 3, and 24 h of induction by tetracycline. Bar = 10 μm. (B) Representative immunoelectron image of a trophozoite expressing HA-EHD1 24 h after tetracycline induction, stained with 15-nm gold–anti-HA. c, cytosol; MVB, multivesicular body; ILV, intraluminal vesicle. Bar = 200 nm.

We also expressed HA-EHD1 under the control of tetracycline (Tet) induction. IFA analysis of HA-EHD1 showed that the protein is similarly localized to membranes of various vesicles after 1 h and 3 h of Tet-induced expression (Fig. 7A, left and middle, respectively). However, at 24 h after induction with Tet, we noticed drastic changes in the localization as well as in the overall intracellular vesicular patterns of expressing trophozoites (Fig. 7A, right), wherein large multivesicular bodies (MVBs) that were also marked with anti-HA signal were observed (Movie S1). This phenotype was exhibited by 32.4% of expressing cells (*n* = 105) that showed MVBs with diameters ranging from 5 μm to 14 μm when measuring from one of multiple confocal planes. These findings were also supported by immunoelectron micrographs, showing immunodecoration of gold–anti-HA particles along the membranes of MVBs, including the neck of invaginated vesicles (Fig. 7B), after 24 h of Tet-induced expression of HA-EHD1. These data point to the involvement of EHD1 in the biogenesis of MVBs in E. histolytica.

10.1128/mbio.03849-21.1 MOVIE S1 Multiple z-section images of fixed HA-EHD1, 24 h after tetracycline induction. Green and red signals indicate anti-HA and anti-APSK antibodies, respectively. Bar = 5 μm. Download Movie S1, AVI file, 0.6 MB. Copyright © 2022 Santos et al. 2022 Santos et al. https://creativecommons.org/licenses/by/4.0/ This content is distributed under the terms of the Creative Commons Attribution 4.0 International license.

### EHD1 is involved in early endosome formation during macropinocytosis and receptor-mediated endocytosis.

**FIG 8:**
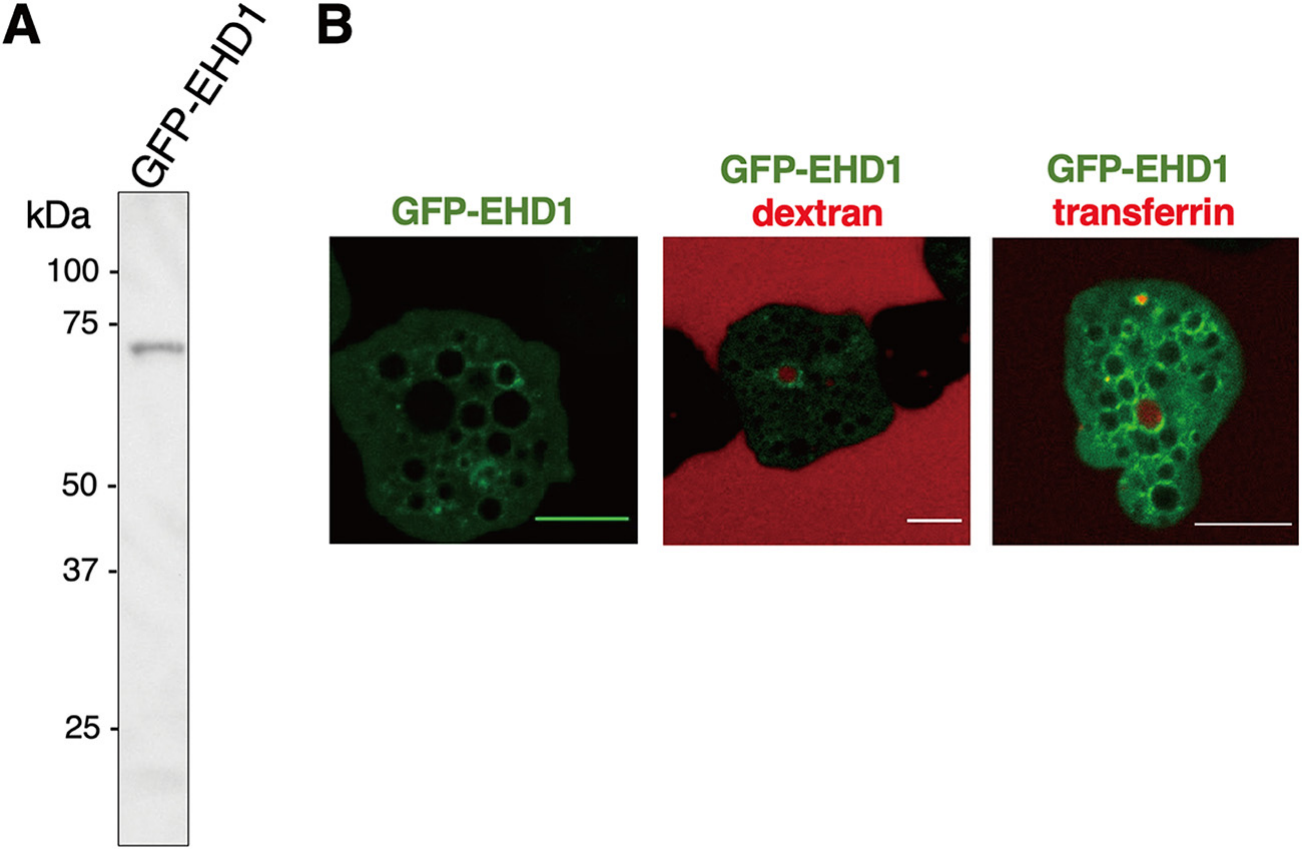
Involvement of GFP-EHD1 in amoebic endocytosis. (A) Anti-GFP immunoblot analysis of approximately 20 μg total lysate of GFP-EHD1-expressing trophozoites. (B) Confocal microscopy images from movies of live trophozoites expressing GFP-EHD1 (left) and GFP-EHD1 in medium supplemented with either RITC-dextran (middle) or Alexa Fluor 568-transferrin (right). Bar = 10 μm.

To further characterize the vesicles whose membranes are associated with EHD1, we performed endocytosis assay using either dextran conjugated to rhodamine B isothiocyanate (RITC) (for bulk endocytosis and macropinocytosis) and to transferrin conjugated to Alexa Fluor 568 (for receptor-mediated endocytosis) as substrates, which were chased by live (green fluorescent protein [GFP]-EHD1 and mock-GFP) or fixed (HA-EHD1 and mock-HA) imaging analysis of treated strains. Expression of GFP-EHD1 was confirmed as a single band after anti-GFP immunoblotting (Fig. 8A). From imaging of live GFP-EHD1-expressing cells, we observed that the GFP-EHD1 signals were evenly spread on round endosomal membranes (Fig. 8B, left). However, signal polarization occurred on portions where there is contact between two endosomes (Movie S2). We also observed localization of GFP-EHD1 in endosomes that contain RITC-dextran and Alexa Fluor 568-transferrin (Fig. 8B, middle and right, respectively). Our analysis also revealed that EHD1 is involved in early endosome formation during macropinocytosis of RITC-dextran. Membranes of newly formed vesicles after ingestion of RITC-dextran initially did not contain GFP-EHD1, but several seconds later, GFP-EHD1 showed an intense signal on the membrane of the enclosing early endosome (Movie S3). Consistent with this, we also noticed a similar phenomenon of GFP-EHD1 recruitment in closing early endosomes when Alexa Fluor 568-transferrin was used as the substrate (Movie S4). In addition, we observed accumulation of transferrin on to certain spots in the plasma membrane which showed remarkably high GFP-EHD1 signals (Movie S5). This suggests that EHD1 is also involved in intravesicular traffic of transferrin with some aggregate signals localized near the PM, likely hinting at its involvement in receptor or membrane recycling.

10.1128/mbio.03849-21.2 MOVIE S2 Live imaging of GFP-EHD1 after 24 of tetracycline induction. Bar = 5 μm. Download Movie S2, AVI file, 2.4 MB. Copyright © 2022 Santos et al. 2022 Santos et al. https://creativecommons.org/licenses/by/4.0/ This content is distributed under the terms of the Creative Commons Attribution 4.0 International license.

10.1128/mbio.03849-21.3 MOVIE S3 Live imaging of GFP-EHD1 trophozoites chased immediately after addition of RITC-dextran. Note the recruitment of GFP-EHD1 in newly closed endosomes. Bar = 5 μm. Download Movie S3, AVI file, 2.8 MB. Copyright © 2022 Santos et al. 2022 Santos et al. https://creativecommons.org/licenses/by/4.0/ This content is distributed under the terms of the Creative Commons Attribution 4.0 International license.

10.1128/mbio.03849-21.4 MOVIE S4 Live imaging of GFP-EHD1 trophozoites chased immediately after addition of Alexa Fluor 568-transferrin. Note the recruitment of GFP-EHD1 in newly closed endosomes. Download Movie S4, AVI file, 3.3 MB. Copyright © 2022 Santos et al. 2022 Santos et al. https://creativecommons.org/licenses/by/4.0/ This content is distributed under the terms of the Creative Commons Attribution 4.0 International license.

10.1128/mbio.03849-21.5 MOVIE S5 Live imaging of GFP-EHD1 trophozoites chased immediately after addition of Alexa Fluor 568-transferrin. Note the accumulation of GFP-EHD1 in the plasma membrane where aggregated transferrin is located. Bar = 5 μm. Download Movie S5, AVI file, 9.8 MB. Copyright © 2022 Santos et al. 2022 Santos et al. https://creativecommons.org/licenses/by/4.0/ This content is distributed under the terms of the Creative Commons Attribution 4.0 International license.

### HA-EHD1 is localized to phagosome and trogosome membrane.

**FIG 9:**
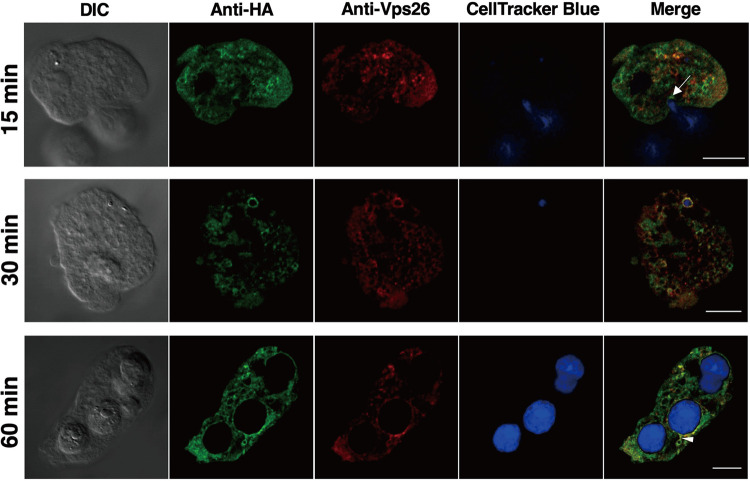
Participation of HA-EHD1 in amoebic phagocytosis and trogocytosis. Representative IFA images of fixed anti-HA (green) and anti-Vps26 (red) double-stained HA-EHD1 trophozoites 15, 30, and 60 min (top to bottom) after coincubation with CellTracker blue-stained CHO cells. The white arrow in the top panel indicates the base of the phagocytic cup. The white arrowhead in the bottom panel points to the tubulation of a trogosome. Bar = 10 uμ

To assess whether amoebic EHD1 also participates in phagocytosis (as well as in trogocytosis), we performed a phagocytosis assay by coincubating expressing trophozoites with CellTracker blue-stained Chinese hamster ovary (CHO) cells. Live- and fixed-cell imaging analyses of phagosomes or trogosomes containing whole CHO cells or bites of CHO cells, respectively, were observed at various time points after coincubation. We observed association of either GFP-EHD1 or HA-EHD1 with some phagosome and trogosome membranes (Fig. 9). We also noticed patches of higher-intensity signals in certain regions of contact between phago- or trogosomes and other vesicles in both fixed-cell (Movie S6) and live-cell (Movie S7) imaging analyses. IFA analysis also suggest that HA-EHD1 is localized at the phagocytic cup/tunnel, suggesting its involvement in early phagosome formation (Fig. 9, top; 15 min after coincubation). Also observed in fixed cells was the localization of HA-EHD1 on the trogosome membrane that appears to undergo tubulation (Fig. 9, bottom; 60 min after coincubation).

10.1128/mbio.03849-21.6 MOVIE S6 Multiple z-section images of fixed HA-EHD1, 60 min after coincubation with CellTracker blue-stained CHO cells. Bar = 5 μm. Download Movie S6, AVI file, 1.5 MB. Copyright © 2022 Santos et al. 2022 Santos et al. https://creativecommons.org/licenses/by/4.0/ This content is distributed under the terms of the Creative Commons Attribution 4.0 International license.

10.1128/mbio.03849-21.7 MOVIE S7 Live imaging of GFP-EHD1 trophozoites chased a few minutes after coincubation with CellTracker blue-stained CHO cells. Bar = 5 μm. Download Movie S7, AVI file, 0.5 MB. Copyright © 2022 Santos et al. 2022 Santos et al. https://creativecommons.org/licenses/by/4.0/ This content is distributed under the terms of the Creative Commons Attribution 4.0 International license.

### Recombinant His-EHD1 demonstrated ATPase activity *in vitro*.

**FIG 10:**
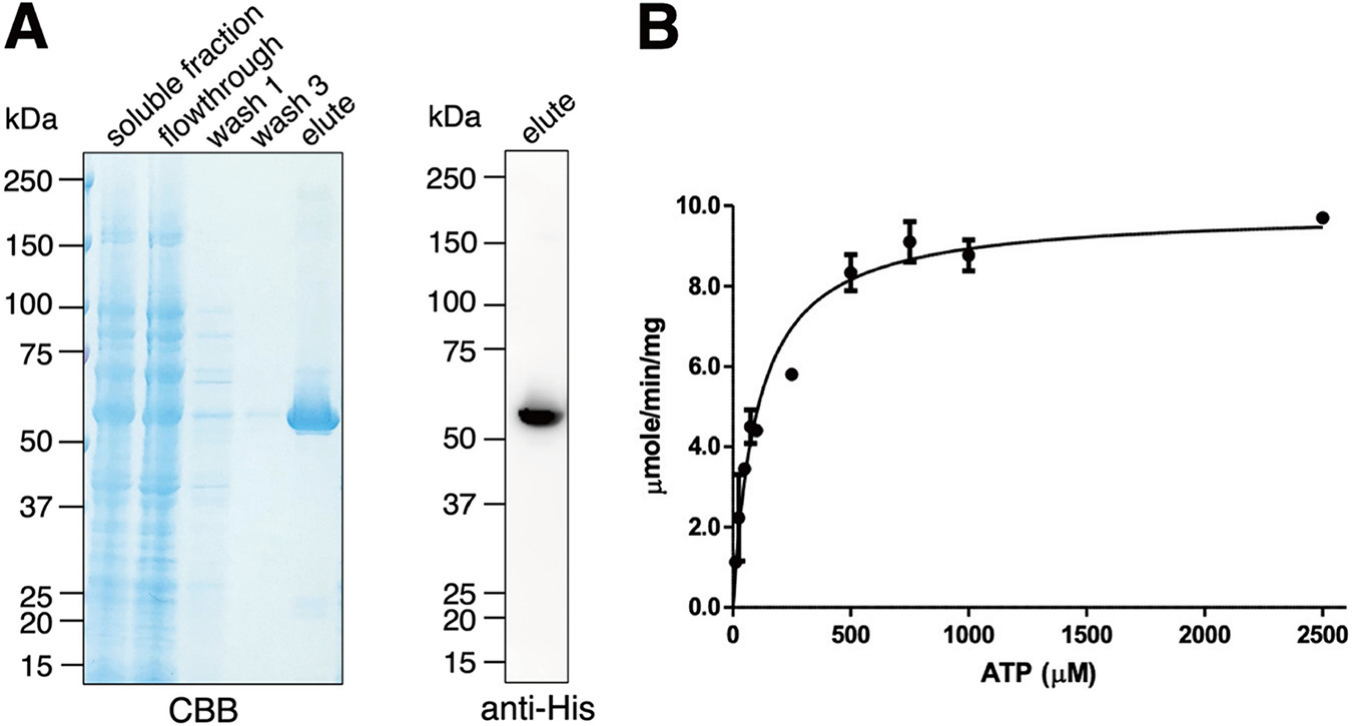
Activity assay of purified recombinant His-EHD1. (A) Coomassie brilliant blue-stained SDS-PAGE gel (left) and anti-His immunoblot (right) of purification fractions of His-EHD1. (B) Specific activity of His-EHD1, determined using ATP as the substrate at various concentrations.

We also expressed amino-terminally histidine (His)-tagged E. histolytica EHD1 in bacteria to assess its enzymatic activity *in vitro*. We purified His-EHD1 using nickel-nitriloacetic acid (Ni-NTA)-agarose beads as shown by the Coomassie brilliant blue-stained SDS-PAGE gel, as well as the anti-His antibody-stained polyvinylidene difluoride (PVDF) membrane (Fig. 10A), containing representative Ni-NTA purification fractions. Eluted fraction of purified His-EHD1 demonstrated ATPase activity (Fig. 10B) with a Michaelis constant (*K_m_*) value of 94.91 ± 16.63 μM and a maximum velocity (*V*_max_) of 9.85 ± 0.37 μmol/min/mg.

## DISCUSSION

We have verified our prediction of ETMP1 being localized to the mitosomal membrane by imaging and fractionation analyses. The gene encoding this protein is essential to the parasite’s proliferation, as indicated by the failure of transfected trophozoites to survive sublethal concentration of drug pressure, compared with those transfected with an empty vector control. Previous attempts at silencing the genes encoding other mitosomal membrane proteins, such as Tom40 (23) and MBOMP30 (24), also failed, suggesting the essential role that these proteins, and the mitosome itself where they exclusively localize, play in the proliferation of E. histolytica. Overexpression of ETMP1 also affected the growth rate of the parasite negatively. This may be due to the disruption of tight regulatory mechanisms for maintaining mitosomal homeostasis and/or formation of toxic protein aggregates. It could also be due to the stoichiometric imbalance of HA-ETMP1-containing protein complexes. Our BN-PAGE analysis identified ETMP1 in the 90-kDa and 180-kDa complexes, whose formation, compositional ratios, and biological functions may be sensitive to ETMP1 overexpression.

HA-ETMP1 immunoprecipitated a unique ∼55-kDa protein. Mass spectrometry analysis of the excised silver-stained gel band indicated several candidates, including EH-domain containing protein (EHD1; EHI_105270; 58 kDa) and its ortholog (EHD2; EHI_152680; 58 kDa) sharing 82% identity, vacuolar protein sorting-associated protein 45 (60 kDa; EHI_154290), and l-myo-inositol-1-phosphate synthase (57 kDa; EHI_070720). Incidentally, when we sequenced the 90- and 180-kDa BN-PAGE complex bands that included HA-ETMP1, we identified EHD1 and its close homolog EHD3 (EHI_052870; 58 kDa) with 97% identity. From these data, we deduced a plausible interaction between ETMP1 and EH domain-containing proteins, with a focus on EHD1 in this paper. Repeated multiple attempts at immunoprecipitating the said complexes failed. One possibility is that the topology of HA-ETMP1 in the complex blocked the HA epitope tag from binding to the anti-HA beads. We also performed IP using HA-EHD1 (Fig. S2B; Table S1D); however, our protein sequencing analysis of the ∼30- to 37-kDa excised band did not detect HA-ETMP1 (Table S1E), suggesting the likely transient nature of this protein binding. The detection of amoebic EHD isotypes in the pulldown and BN-PAGE complexes of HA-ETMP1 suggests potential interaction among these EHD homologs. It is also plausible that amoebic EHDs form heterodimers or hetero-oligomers, as was demonstrated by mouse EHD1 and EHD3. The interaction between mouse EHD1 and EHD3 is likely involved in the regulation of recycling endosomes movement along microtubules (25). In E. histolytica, such EHD oligomers may not only be involved during endocytosis but also exist during the formation and maintenance of the mitosome-endosome contact. Compositional variations of EHD homo- or hetero-oligomers may also exist, and their corresponding functions may be stoichiometry dependent.

EHDs have been associated with roles in various endocytic processes. In one subset known as the C-terminal EHDs, four paralogues are present in mammals, namely, EHD1, EHD2, EHD3, and EHD4. Mammalian EHD1 regulates exit of proteins from the endocytic recycling compartment to the plasma membrane, while both EHD1 and EHD3 have similar roles in controlling early endosome-to-Golgi apparatus transport (26, 27). Mammalian EHD2 localizes to caveolae and, together with the Bin/Amphiphysin/Rvs (BAR)-domain containing binding partner PACSIN2, stabilizes caveolae at the cell surface (28), whereas mammalian EHD4 facilitates macroendocytic uptake of tropomyosin receptor kinase (Trk) receptors (29). EHDs are also implicated in the regulation of endocytic pathways associated with lipid metabolism. Mammalian EHD1 is involved in cholesterol homeostasis, affecting generation of cholesterol and triglyceride lipid bodies (30).

EHDs also regulate endocytosis in other organisms, including plants, worms, and protozoans. Arabidopsis thaliana has two EHD paralogs, *At*EHD1 and *At*EHD2. Downregulation of *At*EHD1 led to a deficiency in the entry of endocytosed material into plant cells, whereas overexpression of *At*EHD2 had an inhibitory effect on endocytosis, suggesting that both proteins are important components in plant endocytic machinery (31). The EHD ortholog in Caenorhabditis elegans, receptor-mediated endocytosis 1 (Rme1), localizes to the endocytic recycling compartment and mediates the exit of cargo proteins to the cell membrane (32). In the protozoan parasite that causes malaria, Plasmodium falciparum, a single EHD protein is encoded in its genome. *Pf*EHD is involved in endocytosis and plays a role in the generation of endocytic vesicles at the plasma membrane that are subsequently targeted to the neutral lipid generation/storage site localized near the food vacuole (33). In the free-living amoebozoan Dictyostelium discoideum, a single gene encoding EHD protein was identified. *Dd*EHD was determined to be involved in phagosome maturation, and its deletion resulted in defects in intraphagosomal proteolysis and acidification, early delivery of lysosomal enzymes, and fast retrieval of the vacuolar H^+^-ATPase in maturing phagosomes (34).

We have shown that *E. histolytica* EHD1 is involved in various endocytic processes. Our live-imaging analysis showed its involvement of in early endosome formation, particularly during closure of newly formed endosomes after engulfment of either RITC-dextran (Movie S3) or Alexa Fluor 568-transferrin (Movie S4), suggesting that *E. histolytica* EHD1 may participate in the scission of early endosomes generated from macropinocytosis as well as receptor-mediated endocytosis. Vesicle tubulation and scission are associated roles of EHDs, due to the presence of the dynamin-like ATPase domain, as demonstrated previously (34–37).

Our *in vitro* enzyme assay showed that His-EHD1 has ATPase activity with a *K_m_* value of 94.91 ± 16.63 μM compared to the previously reported *K_m_* values for mouse EHD1 (80 μM) and *Ce*RME1 (30 μM) (38). We also attempted to investigate the role of ATPase activity of EHD1 in E. histolytica by expressing an ATPase-deficient dominant negative mutant; however, the transfectants did not survive drug selection, suggesting the importance of EHD1 ATP hydrolysis in amoebic biology. Based on other works, ATPase activity of C-terminal EHD-containing proteins is crucial for various stages of the endocytic traffic machinery. Hydrolysis of ATP was essential for binding of human EHD2 complexes to caveolae during clathrin-independent endocytosis (39). It is suggested that membrane scission results from ATP hydrolysis by human EHD2 *in vivo* (35). Using cross-complementation assays in C. elegans, it was found that ATP binding and hydrolysis of human EHD1 are essential for endocytic recycling. It was also shown using *in vitro* liposome-based assays that ATP binding of human EHD1 promotes scaffold self-assembly, while ATP hydrolysis enables extension of bulges and thinning of tubular model membranes, which leads to scission (40). *In vitro* analysis also revealed that ATP binding and concomitant hydrolysis allow membrane remodeling into highly curved tubules (29). We can only hypothesize that ATP hydrolysis in amoebic EHD1 may have functions similar to those of its homologs in other organisms.

We also detected *E. histolytica* EHD1 in the phagocytic cup, and membranes of phagosomes and trogosomes, although only a few phagosomes and trogosomes are labeled with either GFP-EHD1 in live, or HA-EHD1 in fixed imaging analyses. The same can also be said when we performed an endocytosis assay using either RITC-dextran or Alexa Fluor 568-transferrin. This suggests that the nature of EHD localization is dependent on either recruitment by interacting proteins or association/binding with certain lipids on vesicular membranes at specific time points. This is reflected by the localization of either GFP-EHD1 or HA-EHD1 in membranes of vesicles of various sizes and the seemingly polarized signal intensity at sites where two vesicles are in close contact.

As suggested by our lipid overlay assay result, amoebic EHD1 preferentially binds to PI(3,5)P_2_ and PI(4,5)P_2_. PI(4,5)P_2_ has been demonstrated to be localized to the plasma membrane (41), lipid rafts, and uroids (42) of E. histolytica. It is important to note that PI(4,5)P_2_ localized at the plasma membrane is involved in initiating internalization during endocytosis, micropinocytosis, and phagocytosis (43, 44), whereas PI(3,5)P_2_ has a critical role in endosome/lysosome biogenesis and in the initiation of MVB formation (45). Together, these results circumstantially support our observations of amoebic EHD1 localization and involvement in early endosome, intraluminal vesicle, and MVB formation.

Regarding the possible role(s) of mitosome-endosome contact in E. histolytica, we posit that this MCS may be involved in lipid transfer, ion transport, and quality control. Lipid transport and/or metabolism are roles commonly ascribed to MCSs. Although we did not detect any lipid transport proteins in our immunoprecipitation assay, two lipid transport proteins (LTP1 and LTP3) in E. histolytica have been characterized (46), and it is plausible that various LTPs may transiently interact with amoebic MCSs to facilitate lipid mobility across organelles. We detected a few fatty acid ligases in the ∼90- and ∼180-kDa complexes; however, the interaction of these proteins to the HA-ETMP1-containing complex needs to be experimentally validated. Future characterization of these amoebic LTPs and fatty acid ligases, coupled with lipidomic profiling of organelles, including mitosomes and endosomes, will provide clues as to the nature of lipid exchanges that may occur within the MCSs in E. histolytica.

Alternatively, ion transport may also be facilitated in this MCS, as was demonstrated in epithelial cells, where the mitochondria and endosomes that contain iron-bound transferrin are involved in “kiss-and-run” interactions, leading to iron transfer from endosomes to mitochondria (47). It remains to be tested, however, if a similar mechanism involving the mitosome-endosome contact site is used for the transport of key substrates of the sulfate activation pathway (e.g., sodium, sulfate, and phosphate ions) in lieu of still-uncharacterized outer membrane transporter(s). Another possibility is the involvement of EHD1 in mitosomal dynamics. Mitochondria undergo dynamics of fusion and fission to ensure maintenance of homeostasis, control of reactive oxygen species, apoptosis, and autophagy. Dynamin and dynamin-related proteins (Drps) have been implicated in mitochondrial fission. Recently, in HeLa cells, EHD1 was reported to be a novel regulator of mitochondrial fission via a mechanism distinct from that of dynamin/Drp. In this model, human EHD1, together with its binding partner rabankyrin-5, interacts with the retromer complex to participate in mitochondrial division. EHD1 was suggested to facilitate the fission of vesicles that transport Vps35, a retromer complex component, from endosomes to the mitochondrial membrane. It was also suggested that Vps35 may interact with inactive Drp1 on the mitochondrial membrane, causing its removal and subsequent action of active Drp1 to perform mitochondrial fission (48). Fission has also been reported in MROs of anaerobic parasites such as the hydrogenosomes of Trichomonas vaginalis and the mitosomes of E. histolytica (7, 49, 50). Mitosome fission in E. histolytica involves a heterodimer complex of two dynamin-related proteins, DrpA and DrpB (50). It will be interesting to determine if amoebic EHD1 also takes part in influencing mitosome fission, as was postulated for mammalian cells (48).

An alternative novel pathway for mitochondrial quality control that is independent of autophagy protein 5 (Atg5) and microtubule-associated protein 1 light chain 3 (LC3) is the formation of mitochondrion-derived vesicles targeted to lysosomes. Ultrastructural analysis of COS7 cells identified the presence of vesicles that are Tom20 positive within MVBs (51). Furthermore, in hepatocytes, a complex made up of EHD2, EH domain-binding protein 1 (EHBP1), and Rab10 promotes extension of the LC3-containing autophagic membrane in order to engulf lipid droplets during lipophagy (52). Such related pathways may also exist in E. histolytica, and this possibility warrants further investigation.

### Conclusion.

We report a novel membrane contact site between mitosomes and endosomes of Entamoeba histolytica. This unprecedented MCS features the mitosomal membrane protein ETMP1 and a C-terminal EH domain-containing protein, EHD1. ETMP1 is a protein unique to *Entamoeba* and is essential to parasite proliferation. It interacts with EHD1, a protein involved in various endocytic processes in E. histolytica, namely, in early endosome formation during bulk and receptor-mediated endocytosis, in phagocytosis and trogocytosis of mammalian cells, and in the invagination of intraluminal vesicles for the generation of multivesicular bodies. This novel ETMP1-EHD1 interaction hints at a possible role of this mitosome-endosome MCS in various physiological processes that have been demonstrated in other organisms. We thus propose that the ETMP1-EHD1-mediated contact site is involved in lipid transfer, biogenesis, autophagy, organelle dynamics, and quality control of MROs. Further investigation is needed to fully dissect the molecular mechanisms and functions of this and other MRO-related MCSs.

## MATERIALS AND METHODS

### Entamoeba histolytica cultivation.

Entamoeba histolytica HM-1:IMSS strains Cl6 (53) and G3 (54) were maintained in Diamond’s BI-S-33 medium (53) as described previously. Subculturing was performed after incubation for up to 3 to 4 days when trophozoites reached the late logarithmic phase.

### Plasmid construction.

Extraction of total RNA from E. histolytica trophozoites, purification of mRNA, and synthesis of cDNA were performed by following protocols described previously (24). For the expression of hemagglutinin (HA)-tagged proteins in E. histolytica trophozoites, target genes (*etmp1* [EHI_175060] and *ehd1* [EHI_105270]) were amplified by PCR using E. histolytica cDNA as the template and the corresponding primer sets: etmp1-XmaI-fwd, GTTcccgggATGGAACAAATAACTGAAGAA; *etmp1*-XhoI-rev, GAActcgagTTATTTTTTCATTTTTCTTAAGG; and *ehd1*-XmaI-fwd, GTTcccgggATGTTTGGTAAGAAGAAACAAAAACC; *ehd1*-XhoI-rev, GAActcgagTTATTCAACTGGTGGAAGATTGTC (lowercase letters indicate restriction recognition sequence). These PCR amplicons were inserted into plasmids pEhEx-HA and pEhEx-GFP for constitutive expression (55) and pEhtEx-HA and pEhtEx-GFP for tetracycline-induced expression (50), after digestion with XmaI and XhoI (New England Biolabs, Beverly, MA, USA), and then ligated using a ligation convenience kit (Nippongene, Tokyo, Japan). For the expression of recombinant proteins in Escherichia coli, PCR-amplification of *ehd1* was performed using E. histolytica cDNA as the template and the primer set *ehd1*-BamHI-fwd (GTTggatccATGTTTGGTAAGAAGAAACAAAAACC) and *ehd1*-SalI-rev (GAAgtcgacTTATTCAACTGGTGGAAGATTGTC). Digestion and ligation to BamHI- and SalI-linearized plasmid pColdI (TaKaRa, Shiga, Japan) were performed. For transcriptional gene silencing, ∼400-bp fragments of *etmp1* and *ehd1* were amplified using cDNA and the primer sets etmp1gs-StuI-fwd (GTTaggcttATGGAACAAATAACTGAAG)–etmp1gs-SacI-rev (GAAgagctcCTAATTTGATTCCTTTTAAAG) and ehd1gs-StuI-fwd (GTTaggcctATGTTTGGTAAGAAGAAACAA)–ehd1gs-SacI-rev (GAAgagctcTAAATTTAGCCATAAATTCAT). The amplicons were digested with StuI and SacI and ligated to pSAP2-Gunma (56).

### Amoeba transfection and drug selection.

The constructed plasmids described above were transfected by lipofection into E. histolytica trophozoites, as described previously (57, 58). Selection of transfectants was performed by changing the culture medium supplemented with G418 (Gibco/Life Technologies, USA) for those transfected with pEhEx-based and pSAP2-based plasmids or with hygromycin (Fujifilm Wako, Japan) for those transfected with pEhtEx-based plasmids. The starting concentration of 1 μg/mL for added G418 or hygromycin was gradually increased until all control cells (transfected without plasmid) died from the antibiotic challenge. All resultant strains were maintained in medium containing 10 μg/mL G418 or 20 μg/mL hygromycin, unless otherwise stated. For tetracycline induction of protein expression, 10 μg/mL tetracycline was added to semiconfluent cultures 24 h prior to performing assays, unless otherwise stated.

### IFA.

A double-staining immunofluorescence assay was performed as previously described (13), using anti-HA mouse monoclonal antibody (clone 11 MO; Covance, USA) diluted 1:500 in 2% saponin and 0.1% bovine serum albumin in phosphate-buffered saline (saponin-BSA-PBS), to detect HA-tagged ETMP1 and EHD1, respectively, and one of the following polyclonal rabbit antisera diluted in saponin-BSA-PBS anti-adenosine-5′-phosphosulfate kinase (APSK; EHI_179080; a mitosomal matrix protein [56]) diluted 1:300, anti-vacuolar protein sorting 26 (Vps26; EHI_062490; a retromer complex component diluted 1:500 [19]), anti-Rab11B (EHI_107250; involved in cysteine protease secretion [22] diluted 1:500), and anti-pyridine nucleotide transhydrogenase (PNT; EHI_014030; a novel class of lysosomal PNT diluted 1:100 [21]). Secondary antibodies used were Alexa Fluor 488–anti-mouse antibody and Alexa Fluor 568–anti-rabbit antibody (Thermo Fisher) diluted 1:1,000 in saponin-BSA-PBS. Cells were visualized using an LSM780 (Carl Zeiss Microscopy, Germany) confocal laser scanning microscope. At least two trials were performed. Pearson correlation analysis of at least 30 expressing cells per strain was carried out using Zen software (Carl Zeiss, Germany). Profiles of fluorescence intensities along the line were obtained using ImageJ (59).

### Subcellular fractionation and immunoblot analysis.

Trophozoites at the late logarithmic phase were collected and washed three times with 2% glucose–PBS. Cells were mechanically disrupted using a Dounce homogenizer as described previously (13). The resulting homogenate was separated by Percoll gradient fractionation as previously described (13, 18). For carbonate fractionation, organelle-enriched fractions from HA-ETMP1, MBOMP30-HA, HA-EHD1, and mock control homogenates were collected by centrifugation at 100,000 × *g* for 60 min at 4°C. The resultant pellet was reacted with sodium carbonate as previously described (13, 23, 24). All fractions collected were run in SDS-PAGE followed by Western blotting as previously described (60). Immunostaining of PVDF membranes was performed using the following primary antibodies diluted 1:1,000 in 0.1% Tween 20-Tris-buffered saline unless otherwise stated: anti-HA antibody, anti-APSK antiserum (organelle fraction marker), anti-cysteine synthase 1 (CS1; EHI_171750; cytosolic enzyme involved in cysteine metabolism) (61), and anti-cysteine protease binding family protein 1 (CPBF1; EHI_164800, membrane fraction control) diluted 1:100 (62), and chemiluminescent bands were visualized using an LAS-4000 mini luminescent image analyzer (Fujifilm Life Science, Tokyo, Japan). All fractionation experiments were performed at least twice to assess data reproducibility.

### *In silico* predictions and analyses.

Transmembrane domain-containing mitosomal proteins were predicted using a pipeline developed in our previous study (12). To search for homologs of ETMP1 in various *Entamoeba* species, we used as a query the E. histolytica protein EHI_175060 and implemented a BLAST search using the *Amoebozoa* resource database, AmoebaDB (63). Coiled-coil regions were predicted using DeepCoil (64).

### Immunoelectron microscopy.

Samples were prepared as described previously (24). The specimens were double-stained with anti-HA mouse antibody and anti-APSK rabbit antiserum (56). Processing and visualization were performed by Tokai Microscopy, Inc. (Nagoya, Japan), using a transmission electron microscope (JEM-1400 Plus; JEOL Ltd., Japan) at an acceleration voltage of 80 kV. Digital images with a resolution of 2,048 by 2,048 pixels were taken using a charge-coupled device (CCD) camera (Veleta; Olympus Soft Imaging Solution GmbH, Germany).

### IP of HA-ETMP1 by anti-HA antibody.

Organelle-enriched fractions from HA-ETMP1 and mock pEhEx-HA control homogenates were prepared, and approximately 2 μg of proteins was solubilized per μL of 2% digitonin in IP buffer containing 50 mM bis-Tris-HCl (pH 7.2), 50 mM NaCl, 0.001% Ponceau S, and 10% (wt/vol) glycerol for 30 min on ice. The solubilized fraction was collected by centrifugation at 20,000 × *g* for 30 min at 4°C. Immunoprecipitation was performed as previously described (23). Bound proteins were eluted overnight using 60 μg HA peptide. Eluted fractions were loaded on SDS-PAGE gels, followed by immunoblotting using mouse anti-HA antibody. Silver staining was performed using the silver stain MS kit (Fujifilm Wako Pure Chemical Corporation, Osaka, Japan), according to the manufacturer’s protocol. Protein sequencing by liquid chromatography-mass spectrometry analysis was conducted by the Biomolecular Analysis Facility Core, University of Virginia.

### Lipid overlay assay.

As described previously (20), the lysate of the HA-EHD1-expressing strain was used to probe a P-6001 phospholipid membrane strip (Echelon Biosciences, Salt Lake City, UT, USA). The lysate of HA-SNX1 which binds to PI3P (20) was used as a positive control. The strips were washed three times with 0.1% Tween 20 in PBS (PBS-T), followed by reaction with 1:1,000 anti-HA mouse antibody in 3% BSA-PBS for 2 h at room temperature. The strips were washed and incubated with 1:6,000 horseradish peroxidase (HRP)-conjugated goat anti-mouse IgG (Thermo Fisher Scientific, USA) in 3% BSA-PBS for 1 h at room temperature. Finally, the strips were washed and reacted with the Immobilon ECL Ultra Western HRP substrate (Millipore, USA) following the manufacturer’s instructions.

### Endocytosis assay.

Approximately 1 × 10^5^ GFP-EHD1- or mock-GFP-expressing trophozoites in 1 mL BI-S-33 were placed on a 35-mm collagen-coated glass-bottom culture dish (MatTek Corporation, Ashland, MA) for 15 min to allow cell attachment. The medium was removed and replaced with 1 mL of BI-S-33 supplemented with either 2 mg/mL RITC-dextran (molecular weight [MW] = 70 000; Sigma-Aldrich, USA) or 100 μg/mL Alexa Fluor 568-transferrin (Thermo Fisher Scientific, USA). Chase was performed for up to 30 min for live imaging. For IFA, fixation was conducted with HA-EHD1 and mock-HA strains after 0, 30, 60, and 120 min of addition of either RITC-dextran or Alexa Fluor 568-transferrin. Live images were captured using an LSM780 confocal laser scanning microscope (Carl Zeiss Microscopy, Germany), as the cells were being incubated at 35°C using a temperature-controlled stage plate (Carl Zeiss Microscopy, Germany).

### Phagocytosis assay.

A semiconfluent culture of Chinese hamster ovary (CHO) cells, grown in F-12 medium (Sigma-Aldrich, USA) was stained by the addition of 40 μM CellTracker blue (Thermo Fisher Scientific, USA) for 30 min at 37°C. The medium containing excess dye was removed and the cells were washed in 1× PBS followed by treatment with 0.1% trypsin for 5 min at 37°C. The detached cells were collected and washed with 1× PBS three times by centrifugation at 3,000 rpm for 3 min. Stained CHO cells were resuspended in BI-S-33 medium prior to addition to amoeba cells. Cells were coincubated for 15, 30, and 60 min, after which they were fixed for IFA analysis as mentioned above. A parallel setup was prepared for live imaging analysis using GFP-EHD1 and mock-GFP strains.

### Expression and purification of recombinant His-EHD1.

Escherichia coli strain BL21 was transformed using the pCold-His-EHD1 plasmid described above, and the transformants were selected using LB agar containing 150 μg/mL of ampicillin. Isolated colonies were cultured in LB medium with 150 μg/mL of ampicillin and incubated at 37°C with shaking. A 1-L culture was inoculated and incubated in a shaker at 37°C until reaching an optical density at 600 nm (OD_600_) of 0.7. The culture was flash cooled in an ice water bath for 30 min. Induction of protein expression was made by adding 0.5 mM isopropyl-β-d-thiogalactopyranoside (IPTG) to the medium followed by incubation at 15°C with shaking for 24 h. Cells were collected, and protein expression was confirmed by loading the soluble and insoluble fractions in SDS-PAGE, followed by Coomassie blue staining and anti-His immunoblot analysis, as described previously (65). His-EHD1 was purified by binding with Ni^2+^-NTA His-binding slurry (Qiagen, Germany) and eluting with imidazole as described previously (65). Purified His-EHD1 was stored at −80°C with 20% glycerol in small aliquots until use.

### Enzyme activity assay.

Various amounts of purified His-EHD1 (0, 0.125, 0.25, 0.5, and 1.0 μg) were resuspended in assay buffer (20 mM HEPES [pH 7.5], 0.005% Tween 20, 10% glycerol, 1 mM dithiothreitol [DTT], 20 mM NaCl, and 10 mM MgCl_2_) and loaded in triplicate onto independent wells of a 96-well plate. Then, 2 μL of 100 mM ATP was used as the substrate, and distilled water was added to bring the volume of the mixture to 20 μL. Finally, 20 μL of 2× stock solution (66) containing 100 mM Tris-HCl (pH 7.5), 10 mM MgCl_2_, 0.02% Triton X-100, 0.01% BSA, 2 mM glucose, 0.2 mM NADP, 2 u/mL ADP-hexokinase, 2 U/mL glucose-6-phosphate dehydrogenase, 2 U/mL diaphorase I, 0.1 mM resazurin in dimethyl sulfoxide (DMSO), and 20 mM *N*-ethylmaleimide in DMSO, was added, and the plate was incubated at 37°C for 30 min. For determining kinetic parameters, 0, 12.5, 25, 50, 75, 100, 125, 250, 500, 750, 1,000, and 2,500 μM ATP was used to react with 1.5 μg His-EHD1 for 30 min. The fluorescence was measured continuously at excitation and emission wavelengths of 540 nm and 590 nm, respectively, using a SpectraMax Paradigm multimode microplate reader (Molecular Devices, San Jose, CA, USA).
